# Expert Consensus Recommendations on a Biosimilars Value Framework for the Gulf Cooperation Council Countries

**DOI:** 10.1007/s43441-024-00716-4

**Published:** 2024-10-30

**Authors:** Khalid A. Alnaqbi, Ahmed Al-jedai, Mohamed Farghaly, Mohammed A. Omair, Anas Hamad, Fatemah M. A. Abutiban, Ali Al Shirawi, Hanan Al Rayes, Sarah Aldallal, Sahar Fahmy, Steven Simoens

**Affiliations:** 1 Rheumatology Division, Sheikh Tahnoon Medical City and Tawam Hospital, Al Ain, UAE; 2 Internal Medicine Department, College of Medicine and Health Sciences, UAE University, Al Ain, UAE; 3 Emirates Medical Association, Dubai, UAE; 4 Deputyship of Therapeutic Affairs, Ministry of Health, Riyadh, KSA Saudi Arabia; 5 Colleges of Medicine and Pharmacy, Alfaisal University, Riyadh, KSA Saudi Arabia; 6 Department of Health Economics, Dubai Health Authority, Dubai, UAE; 7 Rheumatology Unit, Department of Medicine, King Saud University, Riyadh, KSA Saudi Arabia; 8 Pharmacy Department, National Center for Cancer Care & Research, Hamad Medical Corporation, Doha, Qatar; 9 College of Pharmacy, QU Health, Qatar University, Doha, Qatar; 10 Department of Medicine, Rheumatology Unit, Jaber Al Ahmed Hospital, Kuwait, Kuwait; 11 Sultan Qaboos University Hospital, Muscat, Oman; 12 Department of Medicine, Prince Sultan Military Medical City, Riyadh, KSA Saudi Arabia; 13 Dubai Health Authority, Dubai, UAE; 14 Emirates Health Economics Society, Dubai, UAE; 15 Department of Health, Abu Dhabi, UAE; 16 Department of Pharmaceutical and Pharmacological Sciences, KU Leuven, Leuven, Belgium

**Keywords:** Biosimilars, Value, Adoption, Policy, Gulf cooperation council

## Abstract

**Objective:**

This paper aims to develop a biosimilar value framework with local stakeholders in Gulf Cooperation Council (GCC) countries.

**Methods:**

A convenience sample of ten key opinion leaders from the United Arab Emirates, Kingdom of Saudi Arabia, Kuwait, Oman and Qatar participated in an expert panel meeting in November 2022 that examined factors positively influencing biosimilar adoption in these countries. The discussion was structured around a conceptual biosimilar value framework and an overview of biosimilar policies as derived from a targeted review of the peer-reviewed and grey literature.

**Results:**

The expert panel agreed on a biosimilar value framework for the GCC countries that is founded on trust, cost savings and contextual considerations. They emphasized the importance of launching educational initiatives that build trust in and expand knowledge of all stakeholders about biosimilars. This also includes making stakeholders aware of the various value propositions of biosimilars as an instrument to produce, for example, cost savings. Finally, they stressed that biosimilar adoption is influenced by contextual factors such as incentives and implementation efforts.

**Conclusion:**

Our proposed biosimilars value framekwork is the first set of recommendations in the Arab countries designed to help policymakers and decision-makers promote biosimilar adoption, both in high-income GCC countries and in low- and middle-income countries.

**Supplementary Information:**

The online version contains supplementary material available at 10.1007/s43441-024-00716-4.

## Introduction

Biosimilars are clinically equivalent in terms of safety and efficacy with their reference biologic medications, and they can enter the market once all exclusivities on the reference product have expired [1]. Similar to the steady increase in the market share of biosimilars over the last 15 years in Europe, and to a lesser extent in the United States (US), Gulf Cooperation Council (GCC) countries are embracing biosimilars, as evidenced by a market share increase from 1.0% in 2018 to 4.3% in 2022 [2]. The GCC consists of Bahrain, Kuwait, Oman, Qatar, Saudi Arabia (KSA), and the United Arab Emirates (UAE).

The regulatory refinement, focus on value-based healthcare, enactment of universal health coverage, and promotion of biosimilars have positioned the Middle East and North Africa (MENA) region as a leading pharmaceutical market. The biosimilar market in the MENA region, worth USD 442.5 million in 2020, is projected to exceed USD 623.7 million by 2027 [2]. In 2019, KSA led the market with sales exceeding 1.8 billion USD, followed by the UAE, Egypt, and Algeria, each with approximately 450 million USD [3]. Comparing these values to the global biosimilars market size, the latter was valued at $29.4 billion in 2023 and is expected to reach $66.9 billion by 2028 [4].

Research studies in the GCC about biosimilars have been on the rise. In light of the high prevalence of chronic diseases (such as diabetes mellitus and rheumatoid arthritis) and increasing expenditure on biologics in the Gulf region [5, 6], biosimilars offer an opportunity to create value in the GCC healthcare systems. For instance, a budget impact simulation from a private healthcare payer perspective in Dubai (UAE) calculated that, when all patients with various rheumatologic, dermatologic and gastrointestinal auto-immune diseases would switch from the adalimumab reference biologic to a biosimilar, total costs would fall by around 30% [7]. Similarly, an economic model using decision analysis estimated significant budget savings from adopting adalimumab biosimilar over the originator, with total switching of all patients resulting in the highest savings of 39% in the UAE, KSA, Qatar, Kuwait, and Lebanon [8]. Furthermore, a study conducted in the UAE on HER-2-positive breast cancer patients demonstrated significant cost savings with the use of trastuzumab biosimilar, thereby facilitating patient affordability and access to treatment [9].

Several hurdles in the MENA region need to be addressed to enhance biosimilar adoption. The first challenge is translating regulatory approval into the successful integration of biosimilars within healthcare systems. Europe successfully led the way by actively engaging regulatory agencies, payers, clinicians, and decision-makers. To develop a distinct regulatory pathway, Europe needed to establish a legal framework underpinning the process for granting marketing authorization. Additionally, they provided evidence and decision support mechanisms for clinicians and patients, implemented incentives, and fostered effective competition [10]. Before we began this project, some GCC countries, such as KSA and the UAE started adopting biosimilars in clinical practice. KSA has also partnered with the FDA in the Biosimilars Working Group (BWG), further strengthening international collaboration in this important area [11]. Secondly, several surveys in KSA revealed that, while physicians were generally familiar with biosimilars, they wished to gain a deeper understanding of the place of biosimilars in clinical practice [12–14]. According to a survey in the MENA region, healthcare professionals (HCPs) were hesitant to use biosimilars due to concerns about the manufacturing process and the regulatory assessment of their safety and efficacy [15]. A survey in Jordan indicated that the level of familiarity and expertise with biosimilars varied substantially among pharmacists [16].

Addressing the hurdles in biosimilar adoption and improving HCPs’ understanding of these alternatives is crucial for effective market integration in the MENA region. As part of this integration, tendering practices play a significant role in determining market dynamics and accessibility of biosimilars [12–16]. Tendering can be approached through single-winner or multiple-winner models. Table 1 highlights the key trade-offs between these approaches, with single-winner tenders offering the potential for lower prices but posing higher supply and competition risks, while multiple-winner tenders provide more stability and choice but may not achieve the absolute lowest prices [17–20].

**Table 1:** The key trade-offs between single-winner and multiple-winder tendering

Criteria	Single-Winner Tenders	Multiple-Winner Tenders
Pricing	• Can achieve the lowest possible prices through intense competition among bidders • Some competing pharmaceutical companies may reduce their drug price but have to wait until the contract ends with the other company	• May not achieve the absolute lowest possible prices compared to single-winner tenders
Supply Reliability	• Increases the risk of drug shortages if the single supplier experiences production or distribution delays	• Maintains a competitive market with multiple suppliers, reducing the risk of shortages
Therapeutic Choice	• May not ensure the optimal therapeutic choice for all patients if the selected product is not fully equivalent in terms of bioavailability, effectiveness, and safety	• Allows for selection of products that best meet the therapeutic needs of diverse patient populations • May encourage multiple non-medical switching depending on the cost-saving
Market Competition	• Can lead to the evolution of a monopoly in the long run, reducing competition and innovation	• Promotes long-term sustainability of the pharmaceutical market and supply • Promotes competition e.g. price, quality and provided service
Procurement Process	• Simplifies the procurement and contracting process for the buyer	• Adds complexity to the procurement and contracting process for the buyer
Corruption Risk	• Higher incentives for corruption in countries with limited procurement traditions	• Lower risk of corruption compared to single-winner tenders in some contexts

Building on the discussion of tendering practices, it is essential to consider the broader value of biosimilars within healthcare systems. Earlier studies have identified several factors that positively influence the adoption of biosimilars, highlighting their role in alleviating drug shortages both locally and globally [3, 21, 22].

The increased advancement and manufacturing of biosimilars not only contribute to supply stability but also generate direct cost savings, either through lower list prices or by prompting price reductions in reference products and competing biosimilars. This, in turn, enhances patient access, alleviates pressure on scarce healthcare resources, and promotes healthcare sustainability [3, 23].

For instance, the adoption of biosimilars in the European Union (EU) has led to a 44% increase in patient access across key markets like France, Germany, Italy, Spain, and the UK [24].

Beyond cost savings, biosimilars drive competition with originators, pushing the latter to explore new indications to extend patents. Additionally, biosimilars often offer improvements in terms of lower immunogenicity, higher stability, and easier delivery modes, which contribute to significant advancements in healthcare [3, 23].

To maximize these benefits, value frameworks are essential in evaluating biosimilars based on clinical effectiveness, safety, cost-effectiveness, and overall impact. These frameworks guide stakeholders—including healthcare providers, payers, and policymakers—in making informed decisions about the adoption and reimbursement of biosimilars. Importantly, value frameworks vary by country and are tailored to the specific needs of each healthcare system.

The aim of this study is to propose a biosimilar value framework for the GCC countries driven by an expert panel meeting with local stakeholders. Informed by a literature review, expert panel discussions were guided by an overview of biosimilar policies in the region and a conceptual biosimilar value framework was developed.

The objectives are to:

1. Identify barriers and facilitators for the commercialization and adoption of biosimilars in the GCC from the perspectives of payers, policymakers, and physicians.
2. Recommend modifications and development of program design and policies to support the demonstration of the value of biosimilars for payers, physicians and patients in the GCC.
3. Develop a GCC biosimilar value framework endorsed by the expert panelists.

## Materials and Methods

### Selection of Countries

This study employed a quantitative research design utilizing face-to-face surveys to validate a biosimilars value framework for the GCC constructed based on an extensive literature review. Survey questions were administered to participants during an expert panel discussion. This study focused on the UAE, KSA, Kuwait, Oman and Qatar. This study focused on the UAE, KSA, Kuwait, Oman, and Qatar, chosen for their varying levels of biosimilar experience and adoption.

### Literature Review

Two experienced researchers conducted a focused, non-systematic review of peer-reviewed and grey literature in PubMed, the conference abstract database of the International Society for Pharmacoeconomics and Outcomes Research (ISPOR), and Google Scholar. The search terms were related to biosimilars, the name of the GCC country, and value or policy aiming to retrieve records published from 2007 until 2022. Publications were selected based on the relevance of their titles and/or abstracts, and the bibliography of relevant records was also searched. Each researcher independently reviewed all retrieved literature and resolved any discrepancies through discussions. Relevant literature informed the development of a conceptual biosimilar value framework, which the research team used as a discussion guide for the expert panel meeting.

**Fig. 1:**
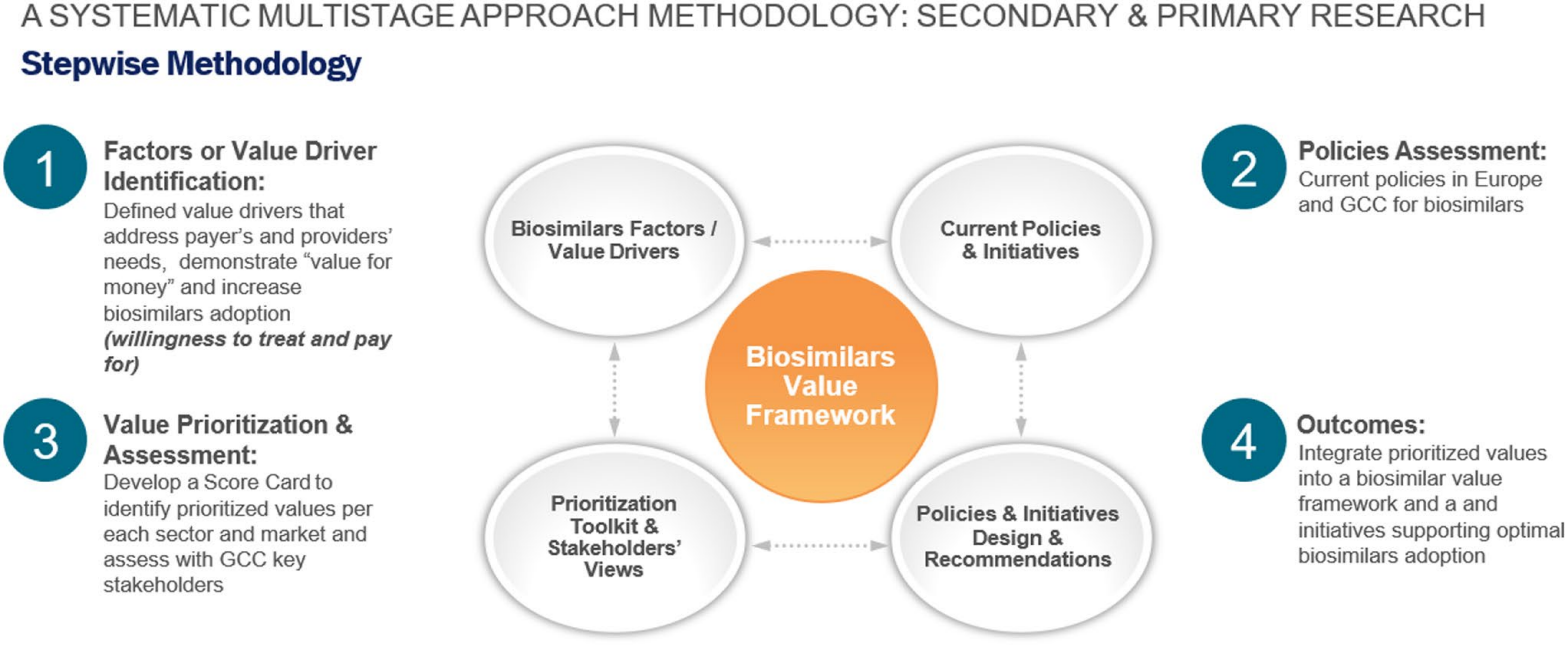
GCC Biosimilar Value Framework Stepwise Methodology

Figure 1 displays the systematic multi-stage methodology, based on primary and secondary research, supporting the development of the GCC biosimilars value framework. The literature review described above identified the value drivers of biosimilars and the current related policies in Europe and the GCC.

### Expert Panel Meeting

An expert panel meeting with senior key opinion leaders from selected GCC countries was held in Dubai (UAE) in November 2022. A convenience sample of experts representing HCPs was composed of decision makers, physicians, health economists, and pharmacists who held professional roles in one or more of the following organizations in the Gulf region: Ministry of Health, regulatory authority, professional society, health insurance company, hospital, or university. A total of ten key opinion leaders from the GCC countries participated in the expert panel meeting, see Table 2. The larger representation from the UAE and KSA reflects their market share, more experience and established biosimilar practices and policies compared to other GCC countries. The expert panel meeting focused on discussing the values and prospects of biosimilars in GCC countries. To validate the framework, the panel was asked to answer seven questions that assessed the perceived impact of various concepts and sub-concepts (elements) on biosimilar adoption. Each element was rated on a scale of 1 to 5, with 1 representing minimal impact and 5 representing significant impact (see Appendix S1). Responses were collected through the polling application Slido (see Appendix S2), ensuring a streamlined and interactive data-gathering process. These questions were at the core of discussions aimed at developing recommendations for a framework and implementation to enhance the adoption of biosimilars in the GCC.

**Table 2:** Profile of expert panel representing GCC countries and their involvement in decision-making and prescribing

Role / Title	Country	Decision Making *	Prescribing role
Pharmacist	KSA	Yes	No
Physician	KSA	No	Yes
Physician	KSA	No	Yes
Physician	Kuwait	No	Yes
Physician	Oman	No	Yes
Pharmacist	Qatar	Yes	No
Physician and Health Economist	UAE	No	No
Physician and Health Economist	UAE	No	No
Physician	UAE	Yes	Yes
Pharmacist	UAE	Yes	No

* Playing an essential role in decision-making on the inclusion of biosimilars in the national formulary and in setting criteria for their use within the hospital setting

Discussions were analyzed following the steps of the Qualitative Analysis Guide of Leuven (QUAGOL) guide [25].

## Results

### Biosimilar Policies

This section provides an overview of biosimilar policies implemented in the GCC countries as published in the literature until 2022.

Neither research and development nor manufacturing policies specific to biosimilars have been implemented in the selected GCC countries. However, some countries have in place general provisions (thus also applicable to biosimilars) that favor local production. KSA, for example, has set a domestic manufacturing target of at least 40% of medicines [26].

Regulatory approval policies for biosimilars vary across the selected GCC countries. For example, when this project began, Oman did not have a clear pathway for approving biosimilars. Similarly, Kuwait and Qatar had not yet introduced specific regulatory approval pathways for biosimilars [27–29]. In contrast, KSA and the UAE have adopted streamlined regulatory processes, offering accelerated assessment and approval for biosimilars [26]. In the UAE, regulatory approval for a biosimilar can be sought two years before the reference biologic’s loss of exclusivity, with final approval granted only after the patent of the reference biologic expires [26].

The Pharmacy and Therapeutics Committee (PTC), composed of multidisciplinary experts, plays an essential role in optimizing pharmacy inventory, thereby enhancing patient care and reducing costs. The inclusion of drugs in formularies is based on the PTC’s evaluation, which considers clinical, ethical, legal, social, logistical, quality-of-life, safety, and economic factors [30]. Each GCC country’s PTC committee follows these criteria in their assessments. A well-managed formulary system aligns the organization’s and the country’s medication-use policies, available therapies, and regularly stocked medications, ensuring cohesion and efficiency. The PTC committee should set policies for prescribing, dispensing, and monitoring biosimilars including interchangeability or switching of biosimilars. Interchangeability describes the ability to substitute one medication with another that is anticipated to produce the same therapeutic outcome [31]. In the GCC countries, unless mandated by the regulatory body or hospital, the decision to switch between the reference product and a biosimilar, or between biosimilars, is left to the prescriber in consultation with the patient. The Department of Health (the regulatory authority in Abu Dhabi, UAE) has recently published a guide to biosimilars for HCPs mandating the use of biosimilars for naïve patients [32]. Similarly, some GCC countries like the UAE, KSA and Qatar utilize formulary management and tendering processes to manage the procurement and reimbursement of biosimilars. It is worth noting that in the Gulf countries, international non-proprietary names (INN; active substances) are not used alone within the formularies of biosimilars. Instead, they are often paired with brand names with specific batch numbers.

Overall, the GCC countries have taken a proactive approach to formulary management of biosimilars, leveraging regulatory frameworks, payment schemes, and procurement processes to expand access to these cost-effective treatments.

Policies about the pricing of biosimilars are in place in two of the selected GCC countries: the UAE and KSA. Both countries set biosimilars prices lower compared to the reference biologic, but with different approaches. The UAE requires a percentage reduction of biosimilars based on the price of the reference biologic, while KSA applies a tiered model, mandatory discounts of at least 25%, 35% and 45% on first, second and third biosimilar, respectively [26, 33].

Procurement policies in the UAE and KSA take the form of direct contracting with providers and tendering [26]. For example, there is generally direct contracting and tendering in the outpatient and inpatient settings in the UAE. A single-winner, national tender is organized every three years in the KSA, but health institutions can also directly contract with manufacturers. Both countries have implemented requirements for sustainable procurement.

The aim of demand-side policies is to increase the uptake of biosimilars in the market, for example, by incentivizing physicians to prescribe biosimilars through prescription quotas; establishing a targeted level for the quantity of prescriptions. These quotas are often accompanied by financial incentives or penalties if not achieved [34]. It is worth noting that, in Abu Dhabi SEHA Company, biosimilars are mandated to be used in inpatient settings. An overview of biosimilar demand-side policies for physicians, pharmacists, and patients in the selected GCC countries is provided in Table 3 which highlights the absence of such policies in these countries, except for clinical guidelines supporting biosimilar prescriptions in the UAE and KSA, and education/information initiatives for HCPs in the UAE, KSA [26], and Qatar. It is noteworthy that neither dispensing policies supporting biosimilar adoption (such as pharmacist substitution or regressive pharmacist margins, where pharmacists’ profit margins are inversely proportional to the medication’s cost) nor policies targeting patients have been adopted in these GCC countries.

**Table 3:**
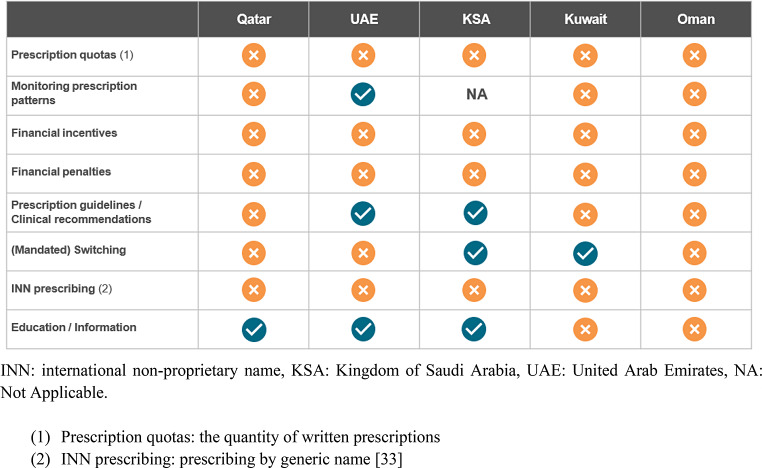
Biosimilar demand-side policies in the Gulf Cooperation Council countries

### Panelists’ Discussion of Biosimilar Value Framework

**Fig. 2:**
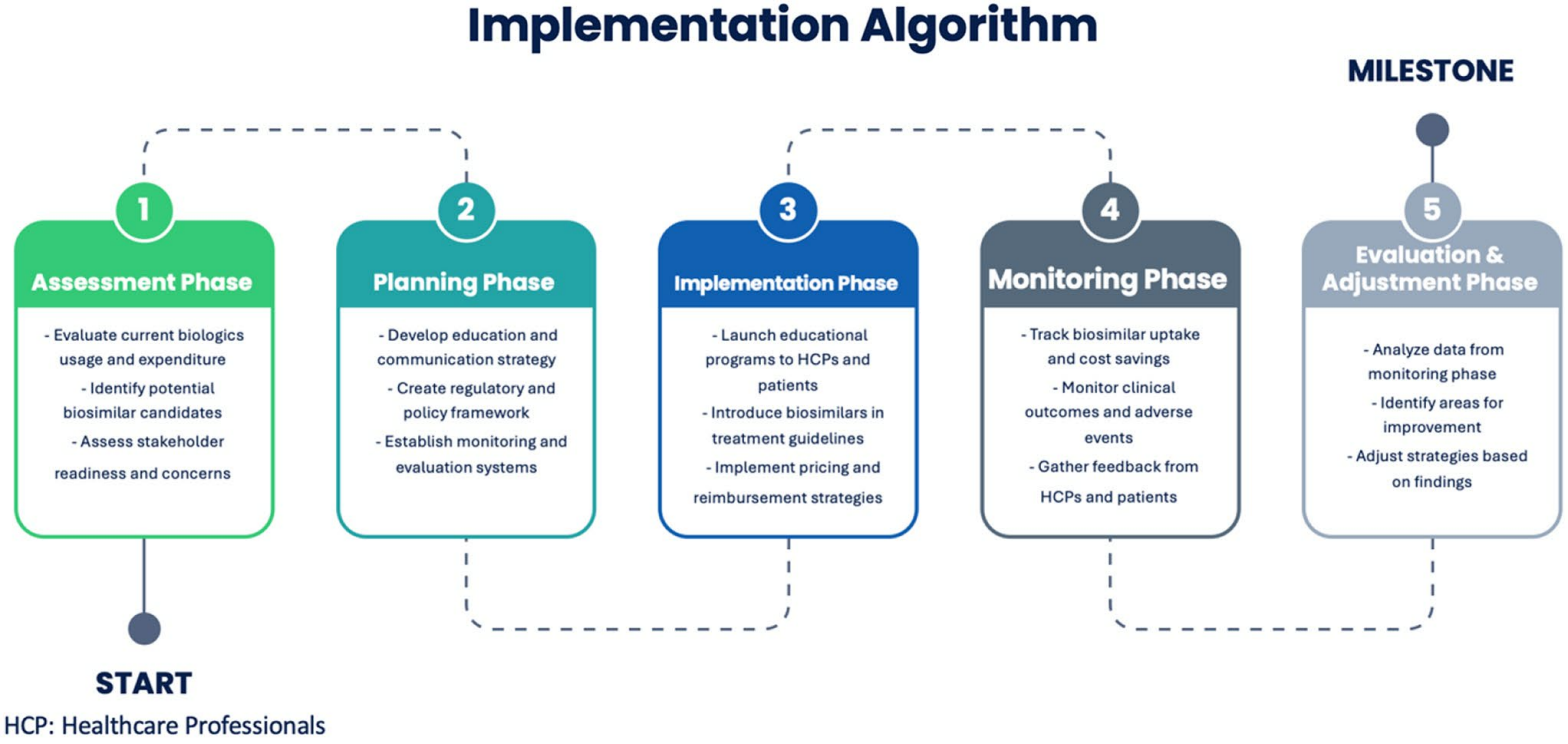
Implementation Algorithm of Biosimilars Adoption and Monitoring in the GCC Countries

Following a roundtable discussion, the panelists developed recommendations for a biosimilars value framework using a prioritization toolkit and incorporating their perceptions and practices (Table 4). Additionally, the expert panel proposed an implementation algorithm for biosimilars adoption and monitoring in the GCC countries (Fig. 2).

**Table 4:** Expert Consensus recommendations for implementing a Biosimilar Value Framework in the GCC countries

Theme	Recommendations
A. Building Trust	1. Clinical Considerations: • Streamline the approval process for biosimilars • Develop treatment guidelines incorporating biosimilars • Encourage the use of biosimilars in biologic-naïve patients • Develop clear guidelines for interchangeability and pharmacy-level substitution • Collect real-world evidence on the efficacy and safety of biosimilars, and switching practices • Follow the international non- proprietary name (INN) for global drug naming consistency
	2. Education and Communication • Develop comprehensive educational programs for healthcare professionals, focusing on biosimilar concepts, misconceptions, regulatory pathways, clinical evidence and real-world data • Create patient-friendly materials explaining biosimilars and their benefits • Establish a multidisciplinary formulary committee in hospitals for optimized biosimilar selection • Enhance communication efforts among all stakeholders for effective collaboration
	3. Monitoring and Evaluation • Conduct regular audits of biosimilar prescribing patterns • Establish a centralized database for tracking biosimilar use, data on immunogenicity, real-world evidence and patient outcome measures • Perform periodic cost-effectiveness analyses • Implement advanced pharmacovigilance tools to report and manage adverse events
	4. Supply Chain Management • Ensure reliable supply of biosimilars • Develop contingency plans for potential shortages • Utilize single/multi-winner tendering to optimize procurement and pricing process
B. Cost Savings:	• Conduct and publish health economic evaluations specific to GCC countries • Develop pricing strategies that ensure significant cost savings compared to reference biologics and/or other biosimilars of the same reference biologics. • Create incentives for healthcare providers and institutions to adopt biosimilars • Allow free drug pricing to foster market competition • Implement mandated discounts to control healthcare costs
C. Contextual drivers	• Enhance strategies to improve patient adherence to therapies • Re-invest biosimilar-generated savings in the healthcare system • Integrate biosimilar policies into national healthcare strategies • Implement a quota system for biosimilar prescribing • Allow healthcare providers to retain a portion of the savings generated from biosimilar use (gain-sharing programs) • Provide CME credits for healthcare professionals attending biosimilar education programs

Experts emphasized that building trust is crucial to driving the adoption of biosimilars across GCC countries. Conversely, the lack of trust among authorities, payers, patients, and caregivers was identified as the primary barrier to biosimilar adoption in Kuwait. This trust can be established through several means, including the assurance that the safety and efficacy of biosimilars are supported by international entities such as the European Medicines Agency (EMA) and the US Food and Drug Administration. Furthermore, trust can be strengthened by drawing on the experience with biosimilars in Western countries, gathering real-world data on immunogenicity and switching practices, and implementing monitoring tools to address any remaining concerns among HCPs and patients. A robust pharmacovigilance strategy including safety specifications for the biologic originator or biosimilar, is an essential driver for uptake and for identifying safety variations. This requires well-defined activities and proposed actions, including an evaluation of potential medication errors. This strategy should also include a risk management plan, providing additional information like medication guide supplements and access restrictions.

Trust can be strengthened further by informing and educating HCPs and patients with a view to raising their understanding of biosimilar concepts and terminology, and dispelling misperceptions [35]. For instance, when biosimilars were introduced in Abu Dhabi SEHA Company, several actions were taken including the distribution of educational materials to patients in some languages such Arabic and English. Additionally, HCPs were invited to attend educational sessions about biosimilars presented by physicians using local cases. This reflected the important role of HCPs in communicating with and reassuring patients. During the expert panel meeting, it was also suggested that the place of biosimilars in clinical practice should be included in treatment guidelines to overcome physician distrust in these products.

When examining future prospects for biosimilars in GCC countries, experts agreed that it is important to set up an ecosystem in which all stakeholders benefit from the adoption of biosimilars. It was argued that such an ecosystem should be founded on a solid regulatory assessment of biosimilars by authorities and should foster competition between manufacturers in the form of lower prices for healthcare payers and the provision of patient support programs that enable HCPs to offer a wider range of medications to patients, with the increased adoption of biosimilars contributing to a viable business model for manufacturers.

Additionally, at the time of the experts meeting, the limited information and regulations in the GCC countries regarding interchangeability hinder switching, which is a significant driver for biosimilar uptake. This switching is often motivated by the need for more suitable patient therapies due to efficacy or tolerability issues with previous biologic products [36].

With respect to tendering, experts discussed the Saudi experience with setting up a single-winner, national tender every three years in the public healthcare system. The result, where one manufacturer supplies the entire market for a relatively long period, is believed in KSA to guarantee low prices, minimize the need to switch patients from one product to another, simplify formulary management in health institutions, and reduce prescribing and dispensing errors. Manufacturers have an incentive to stay on the market as they also have the opportunity to supply the private market (which represents around 25-30% of the total Saudi market). Moreover, the danger of shortages is mitigated by imposing multiple tender conditions, including the need for the manufacturer to keep a stock of at least 30% of the required quantity and to pay for an alternative therapy in case of delayed or cancelled delivery.

Experts agreed with contextual drivers (country-specific considerations), trust and cost savings, as having an impact on biosimilar adoption in the GCC countries. Regarding contextual drivers, it was argued that Oman and Qatar consider it important that policies can be implemented with minimal effort, while KSA prioritizes the introduction of appropriate incentives. All experts emphasized the cost saving potential of biosimilars and, hence, the role that biosimilars can play in providing affordable healthcare to continuously growing populations in GCC countries. Biosimilars were also seen as a key instrument to widen access to healthcare for the large number of patients in the Gulf region. Therefore, it is important to make all stakeholders aware of the specific potential value propositions of biosimilars (e.g. cost savings, increased healthcare access, better treatment adherence, expansion of reimbursed indications) in GCC countries. Given that the importance of biosimilar value propositions may vary between GCC countries and between stakeholders, it was proposed that policies should be tailored to the local setting.

## Discussion

This study has found that the biosimilar ecosystem in the GCC countries is steadily evolving, with some countries, such as the UAE and KSA, adopted biosimilars earlier than others. Moreover, Oman Circular No.115 of 2024 outlines new guidelines for the registration of biological products within the country. These guidelines are part of Oman’s broader efforts to regulate the pharmaceutical sector, ensuring that biological products, including biosimilars, meet stringent safety, efficacy, and quality standards before they can be marketed [37].

Our expert consensus recommendations for implementing a biosimilars value framework in the GCC countries encompass a comprehensive approach to biosimilar adoption and integration into healthcare systems. The recommendations focus on three themes related to building trust, cost savings, and contextual drivers. By addressing these critical aspects, the framework aims to facilitate the successful integration of biosimilars into GCC healthcare systems, leading to improved patient access, cost savings, and overall healthcare quality. This multifaceted approach recognizes the complex nature of biosimilar implementation and provides a roadmap for stakeholders to navigate the challenges and opportunities presented by these innovative therapies.

Improving the efficiency of the approval process through regulatory procedures could greatly speed up the adoption of biosimilars. This could be achieved through collaborative reviews with other regulatory authorities or by utilizing previous expert reviews. Approvals from regulatory bodies could also serve as strong references for these expert reviews [36]. The first consensus-based recommendations on the use of biosimilars in the treatment of inflammatory arthritis in the GCC countries were recently published. One recommendation is that proving a biosimilar’s efficacy and safety for one indication should suffice for its approval in other conditions approved for the reference product. Additionally, other recommendations focused on establishing educational initiatives, national policies to support biosimilar adoption and interchangeability, as well as real-world registries [38].

In all selected GCC countries, it is crucial to build trust in biosimilars by communicating with and educating policymakers, authorities, payers, physicians, pharmacists and patients. This approach addresses any doubts about biosimilars, and also aligns with Europe’s experience and strategy for closing the translational gap in biosimilar integration [10]. As the level of experience with biosimilars is likely to differ between types of stakeholders, the objective and content of these initiatives should be adapted to the needs of individual stakeholder types.

Supporting educational initiatives on biosimilars mirrors the findings of a survey and workshop with stakeholders from the UAE, which stressed the need to disseminate information among physicians to increase biosimilar adoption. Additionally, it was proposed that protocols be implemented to promote the prescription of biosimilars to treatment-naïve patients and that physician prescribing patterns be monitored on a regular basis [39].

Moreover, there is a growing trend of hosting biosimilar conferences in GCC countries. One notable instance is the 2023 International Biosimilars Congress held in Dubai, which featured invited speakers including physicians who prescribe biosimilars, pharmacists, representatives from charitable organizations, and patients [38].

It is noteworthy that the use of biosimilars in clinical trials and its implications for trust, familiarity, and regulation deserve attention. This is an important emerging trend in multi-national trials, with potential impacts on cost reduction and future regulatory strategies. Moreover, this issue represents a key area for future research and policy development in the region as biosimilars become more widely adopted and integrated into clinical practice and research.

When considering factors that influence biosimilar adoption, GCC countries can use the experience of Europe and the US with regulatory assessment, pricing and reimbursement, contracting, and demand-side incentives. The incentives, including those aimed at physicians to decrease costs, can help increase the uptake of biosimilars in the market [40, 41]. For instance, there is hesitancy from prescribers and limited regulatory guidance about the interchangeability of a biosimilar and its reference biologic in GCC countries. This contrasts with Europe where the EMA and the Heads of Medicines Agencies consider biosimilars and their reference biologic to be interchangeable [31] and the US where several biosimilars have been designated to be interchangeable [42]. Furthermore, Alnaqbi et al. 2023 discussed in an international comparative analysis that mandated switches to the lowest-cost alternative, as seen in certain Canadian provinces, may increase biosimilar usage but could limit competition opportunities for other stakeholders. This approach can lead to frequent switching and drive prices down, potentially resulting in unsustainable price levels. Strategies such as prescribing quotas (observed in Germany and the United Kingdom) or financial incentives/penalties tied to biosimilar prescriptions may boost biosimilar adoption in the short term, but do not encourage natural competition between originators and biosimilars [43]. Some GCC countries have experienced interchangeability (non-medical switching) such as the UAE where Abu Dhabi SEHA Company has a policy on biosimilars that encourages physicians to switch stable patients from the reference biologic medication to a biosimilar. At the time of submitting this manuscript, some sectors in the Gulf countries like KSA and Kuwait have started to implement mandatory non-medical switching to biosimilars. This likely followed EMA’s updated 2023 statement approving the interchangeability of biosimilars [31].

The specific characteristics of GCC healthcare systems need to be taken into account when designing a biosimilar ecosystem. For example, European healthcare systems, which tend to be mainly publicly financed, have implemented multi-winner tendering systems in order to promote competitive, but sustainable markets for biosimilars [44]. Alnaqbi et al. 2023 suggested maintaining competition by ensuring the presence of multiple participants. This means that national-level single-winner contracts should be avoided. In cases where single-winner contracts operate at the regional or local level, measures should be implemented to guarantee the existence of multiple suppliers at the national level [43]. However, GCC countries tend to have a sizeable private market in addition to the public healthcare system. While literature comparing tendering systems for biosimilars in these sectors is limited, tendering generally supports the value of multi-tender environments to maintain competition in the market with multiple suppliers, reducing the risk of shortages. In public procurement, relying solely on “price-only” or “winner-takes-all” awards has been increasingly criticized for compromising supply security and competition, as noted by the European Federation of Pharmaceutical Industries and Associations (EFPIA) [45]. Recently, the UAE Cabinet issued Resolution No. (87) of 2023, establishing the Supreme National Committee for Unified Purchasing to oversee the procurement of medicines, medical supplies, and equipment (the ‘Medical Products’) [46].

It is worth studying the comparison between single-winner and multiple-winner tendering in the context of biosimilars in both the private and public markets of the GCC countries. This research could be particularly useful for other countries including low- and middle-income countries. The expert panel considered tendering strategies significant to the success of biosimilar adoption and recommended utilizing a strategy to optimize procurement and pricing, tailored to the needs of each GCC country.

While GCC countries have implemented various policies to capitalize on the competitive advantage of biosimilars, it is important to recognize that the success of these policies can vary. Drawing parallels with the European experience, the somatotropin biosimilar Omnitrope has been used in most European countries for over 15 years, proving to be cost-effective by saving more than $1.9 billion at list price levels across selected European countries between 2006 and 2021. However, the amount of savings differs between countries due to variations in biosimilar adoption, competitive dynamics, and regulatory processes [47]. This highlights the need for continuous evaluation and adaptation of biosimilar strategies in the GCC context. Furthermore, as more physicians in the Gulf become more familiar with prescribing biosimilars, their positive perceptions are likely to improve over time. For example, recent IQVIA data found that an increasing number of physicians in Europe reported more positive perceptions of biosimilars over time as they gained experience with their use [48].

The main strength of this study is its multidisciplinary and comprehensive qualitative analysis of biosimilar values across multiple GCC countries, utilizing available literature and a prioritization toolkit during the expert meeting. Although the limited number of participants in the expert panel meeting is a drawback, we emphasize that our sampling method purported to select high-level opinion leaders who are actively involved in shaping the biosimilar ecosystem in their country and have experience with biosimilar adoption. There was a balanced representation between decision-makers involved in biosimilar adoption and the prescribers themselves. Even though our study contributes to the sparse body of evidence on the biosimilar ecosystem in GCC countries, future research needs to validate our findings and analyze progress with biosimilar adoption in the Gulf region.

## Conclusion

This study proposes recommendations on a biosimilars value framework for GCC countries, focused on promoting trust in biosimilars and pursing cost savings opportunities. Policy and decision-makers need to implement local educational initiatives, provide guidance on interchangeability and monitoring to build trust; and introduce pricing and reimbursement policies to achieve biosimilar cost savings. Additionally, contextual factors influencing biosimilar adoption must be considered. These actionable recommendations aim to create a sustainable biosimilar ecosystem in the GCC region.

## Electronic Supplementary Material

Below is the link to the electronic supplementary material.

Supplementary Material 1

## Data Availability

No datasets were generated or analysed during the current study.
