# Supplementary material for: Expert Consensus Recommendations on a Biosimilars Value Framework for the Gulf Cooperation Council Countries
Source: Ther Innov Regul Sci. 2024 Oct 30;59(1):153–63. doi: 10.1007/s43441-024-00716-4 (PMC11706834; doi:10.1007/s43441-024-00716-4)
Supplement: Supplementary file 1 — Supplementary Material 1 [file 43441_2024_716_MOESM1_ESM.docx]

**Supplementary Material**

**Appendix 1: Poll Survey Questions**

1. On a scale of 1 to 3 (with 3 being the highest), rate the impact of each of the below drivers on biosimilar adoption success
   1. Contextual Drivers
   2. Building Trust
   3. Cost Saving
2. On a scale of 1 to 5 (5 being the highest), rate the impact of each of the below components on biosimilar adoption success
   1. Education and communication
   2. Pricing and reimbursement
   3. Others- contextual considerations
   4. Monitoring
   5. Clear policies and guidance on interchangeability
3. Rate the impact of each of the following Pricing and Reimbursement (P&R) elements on a scale of 1 to 5 (with 5 being the highest positive value) in relation to the success of biosimilar adoption:
   1. Tendering / Single Winner
   2. Free pricing
   3. Mandated fixed discount
   4. Price link (to originator or other biosimilars)
   5. Tendering multi-winner
4. Rate the impact of each of the below monitoring elements on a scale of 1 to 5 (with 5 being the highest positive value), in relation to the success of biosimilar adoption:
   1. INN naming system
   2. Developing data on immunogenicity
   3. Development of patient-specific outcome measures – real-world evidence RWE)
   4. Monitoring the ability to maintain product supply.
   5. Developing monitoring tools for post-commercialization pharmacovigilance measures
5. Rate the impact of each of the below education and communication elements on a scale of 1 to 5 (with 5 being the highest positive value) in relation to the success of biosimilar adoption:​
   1. Understanding biosimilars, market incentives, approval policies, and the rationale for indication extrapolation
   2. Coordinated multi-stakeholder efforts to improve communication between physicians, payers, regulators, patients, and pharmacists
   3. Patient education
   4. A multidisciplinary formulary committee including physicians, pharmacists, administrators, and payers
   5. Defining interchangeability and pharmacy-level substitution rules
6. Rate the impact of each of the following policies on interchangeability, using a scale of 1 to 5 (with 5 being the highest), in relation to the success of biosimilar adoption:
   1. Real-world experience with switching strategies from innovator drugs to comparator (biosimilar) drugs
   2. Clinician approval required for patient switching
   3. Clear regulatory position on interchangeability
   4. Guidance on switching and substitution for interchangeability
   5. Defining interchangeability and rules for pharmacy-level substitution/auto-substitution
7. Rate the impact of each of the following contextual considerations on biosimilar adoption success, using a scale of 1 to 5 (with 5 being the highest):​
   1. Ease of implementing policies
   2. Incentives – quotas, gain sharing, Continuing Medical Education (CME)
   3. Patient benefits – earlier and more suitable treatment
   4. Improved adherence – potentially leading to lower overall healthcare costs
   5. Re-allocation of resources to cover other disease treatments, allowing more patients to be treated

**Appendix 2: GCC Biosimilar Value Framework - Polling results**

On a scale of 1 to 3 (with 3 being the highest), rate the impact of each of the below drivers on biosimilar adoption success

On a scale of 1 to 5 (with 5 being the highest), rate the impact of each of the below components on biosimilar adoption success

Rate the impact of each of the below Price & Reimbursement (R & P) elements on a scale of 1 to 5 (with 5 being the highest positive value) in relation to the success of biosimilar adoption

Rate the impact of each of the below Monitoring elements on a scale of 1 to 5 (with 5 being the highest positive value) in relation to the success of biosimilar adoption

Rate the impact of each of the below Education & Communication elements on a scale of 1 to 5 (with 5 being the highest positive value) in relation to the success of biosimilar adoption

Rate the impact of each of the below Policies on Interchangeability elements on a scale of 1 to 5 (with 5 being the highest positive value) in relation to the success of biosimilar adoption

Rate the impact of each of the below Contextual considerations elements on a scale of 1 to 5 (with 5 being the highest positive value) in relation to the success of biosimilar adoption
